# CXCL9 Is a Potential Biomarker of Immune Infiltration Associated With Favorable Prognosis in ER-Negative Breast Cancer

**DOI:** 10.3389/fonc.2021.710286

**Published:** 2021-08-30

**Authors:** Yuan-ke Liang, Ze-kun- Deng, Mu-tong Chen, Si-qi Qiu, Ying-sheng Xiao, Yu-zhu Qi, Qin Xie, Zheng-hao Wang, Shi-cheng Jia, De Zeng, Hao-yu Lin

**Affiliations:** ^1^Department of Thyroid and Breast Surgery, Clinical Research Center, The First Affiliated Hospital of Shantou University Medical College (SUMC), Shantou, China; ^2^Guangdong Provincial Key Laboratory for Diagnosis and Treatment of Breast Cancer, Shantou, China; ^3^SUMC, Shantou, China; ^4^Clinical Research Center, Diagnosis and Treatment Center of Breast Diseases, Shantou Central Hospital, Shantou, China; ^5^Department of Thyroid Surgery Shantou Central Hospital, Shantou, China; ^6^Department of Hematology, Cancer Research Center Groningen, University Medical Center Groningen, University of Groningen, Groningen, Netherlands; ^7^Department of Medical Oncology, Cancer Hospital of SUMC, Shantou, China

**Keywords:** breast cancer, CXCL9, biomarker, prognosis, immune infiltration

## Abstract

The chemokine CXCL9 (C-X-C motif chemokine ligand 9) has been reported to be required for antitumour immune responses following immune checkpoint blockade. In this study, we sought to investigate the potential value of CXCL9 according to immune responses in patients with breast cancer (BC). A variety of open-source databases and online tools were used to explore the expression features and prognostic significance of CXCL9 in BC and its correlation with immune-related biomarkers followed by subsequent verification with immunohistochemistry experiments. The CXCL9 mRNA level was found to be significantly higher in BC than in normal tissue and was associated with better survival outcomes in patients with ER-negative tumours. Moreover, CXCL9 is significantly correlated with immune cell infiltration and immune-related biomarkers, including CTLA4, GZMB, LAG3, PDCD1 and HAVCR2. Finally, we performed immunohistochemistry with breast cancer tissue samples and observed that CXCL9 is highly expressed in the ER-negative subgroup and positively correlated with the immune-related factors LAG3, PD1, PDL1 and CTLA4 to varying degrees. These findings suggest that CXCL9 is an underlying biomarker for predicting the status of immune infiltration in ER-negative breast cancer.

## Introduction

Breast cancer (BC) is the most prevalent cancer in women worldwide (1). Despite distinct progress in treatment, the oestrogen receptor (ER)-negative subtype of BC still shows difficulty in treatment due to the inadaptability of endocrine therapy. Notably, a variety of clinical trials and studies have suggested that patients with early-stage ER-positive breast cancer may benefit from chemotherapy based on microarray gene expression prognostic tests. However, treatment options for patients with early ER-negative tumours remain limited, and novel treatments are sorely needed (2).

The immune system is involved in breast tumour development, progression, surveillance and elimination (3). At the early stage of BC, acute inflammation occurs and results in both tumour cell obliteration and the tumour-specific T cell response. Eventually, there is a switch from acute to chronic inflammation, constituting a complex tumour microenvironment (TME) consisting of suppressive immune cells that allow immune evasion and tumour development to occur (4). Chemokines, a component of the TME, are known as mediators of immune cell recruitment (5). CXCL9 (C-X-C motif chemokine ligand 9), a selective ligand for CXCR3, is a monokine induced by gamma interferon (MIG) (6). Previous studies have shown that CXCL9 is a favourable prognostic biomarker in patients with solid malignancies and a predictor of better response to anti-PD-1 therapy, while its tumour-promoting effect has also been reported in diffuse large B-cell lymphoma (7–9). CXCL9 has been reported to be associated with promoted intratumour infiltration of immune cells in breast tumours and can be used for predicting therapeutic response (10, 11). However, the exact function of CXCL9 and its association with current immune-related targets in BC remain unknown.

In this study, we aim to investigate the expression pattern of CXCL9 and its relevance to prognosis in distinct molecular subtypes of BC, as well as the features of various tumour-infiltrating immune cells according to the levels of specific marker expression by employing a number of renowned databases, including the Oncomine, Tumour Immune Estimation Resource (TIMER), and Kaplan-Meier Plotter, etc.

## Materials and Methods

### Oncomine Database Analysis

The Oncomine database (https://www.oncomine.org) is a web-based data-mining platform with cancer microarray information comprising 715 datasets and 86,733 samples (12). The level of CXCL9 expression in breast cancer was examined in the Oncomine 4.5 database. The threshold was determined as follows: fold-change of 2 with a P-value of 0.01 and gene ranking of all, as described in our previous study (13).

### GEPIA2 Database Analysis

An updated version of the GEPIA (Gene Expression Profiling Interactive Analysis) database, GEPIA2 (http://gepia2.cancer-pku.cn/), was utilized to pool and analyse the RNA sequencing expression data from the TCGA and GTEx projects, including 9,736 tumours and 8,587 normal samples, using a standard processing pipeline (14). The mRNA expression of CXCL9 in breast cancer and its association with survival outcomes, including disease-free survival (DFS) and overall survival (OS), were examined using GEPIA2.

### bc-GenExMiner Database Analysis

The Breast Cancer Gene-Expression Miner (bc-GenExMiner) (http://bcgenex.centregauducheau.fr) database is a web-based application that offers the evaluation of prognostic value and conducts gene expression correlation analyses in different groups of patients based on 21 public data sets (15). In this study, the bc-GenExMiner database was used to determine the expression of CXCL9 in association with a set of subgroups and estimate the prognostic significance of CXCL9 based on oestrogen receptor status in breast cancer.

### TIMER Database Analysis

The web server of Tumour Immune Estimation Resource (https://cistrome.shinyapps.io/timer/, TIMER) was used to evaluate the abundance of tumour-related immune infiltrating cells, including CD8+ T cells, CD4+ T cells, B cells, dendritic cells, neutrophils, and macrophages. Moreover, the correlation between CXCL9 levels and the abundance of immune infiltrating cells in breast cancer subtypes or specific biomarkers in immunotherapy were analysed by TIMER.

### Kaplan-Meier Plotter Database Analysis

The Kaplan-Meier Plotter (http://kmplot.com/analysis/) is an online website for investigating the potential connection between the expression levels of a number of candidate genes and the survival outcome in breast cancer patients with or without anticancer treatment (13). The association between CXCL9 expression level and survival parameters in breast cancer based on hazard ratios (HRs) and log-rank P-values was assessed with the Kaplan-Meier Plotter.

### GOBO Database Analysis

The Gene Expression-based Outcome for Breast Cancer Online (GOBO) database was used to analyse the survival prognosis of breast cancer patients using the CXCL9 expression level (16).

### UALCAN Database Analysis

The UALCAN database (http://ualcan.path.uab.edu) is an open resource for exploring cancer OMICS data, including TCGA and MET500 (17), allowing the estimation of the level of CXCL9 expression in different molecular subtypes of breast cancer as well as the analysis of the expression of CXCL9 across TCGA cancers.

### UCSC Xena Database Analysis

The UCSC Xena database (http://xena.ucsc.edu/) is a visual tool for exploring both public and private omics data that supports researchers in viewing functional genomics data, such as INDELs, SNVs, DNA methylation, CNV expression, ATAC-seq signals, large structural variants and phenotypic annotations (18). In this study, UCSC Xena was used to perform a survival analysis.

### Human Protein Atlas Database Analysis

The Human Protein Atlas (19) (HPA) database (https://www.proteinatlas.org) is an online tool for systemically investigating the genome-wide transcriptome of the protein-coding genes of 17 major cancer types in terms of survival outcome and to provide diagnostic and prognostic information on the protein expression profiles of a variety of cancers, normal tissues and corresponding cell lines. In this study, survival curves were generated to evaluate the value of CXCL9 in breast cancer prognosis.

### Immunohistochemistry

Breast cancer tissue samples were collected from a series of 115 patients with early-stage breast cancer diagnosed in the Cancer Hospital of SUMC in 2016-2017. The study and related procedures were approved by the Ethical Review Board of the hospital as indicated. Immunohistochemistry was performed as described previously (20). Briefly, slides were deparaffinized and rehydrated, and antigen retrieval was performed. Subsequently, endogenous peroxidase activity was blocked with H_2_O_2,_ and the sections were incubated with primary antibodies against CXCL9 (Abcam, Danvers, MA, USA, 1:200), PD1 (Abcam, Danvers, MA, USA, 1:200), PD-L1 (Abcam, Danvers, MA, USA, 1:200), LAG3 (Abcam, Danvers, MA, USA, 1:200) and CTLA4 (Abcam, Danvers, MA, USA, 1:200) for 1 hour at room temperature (RT). After that, the tissue sections were incubated for 30 min at RT with HRP-conjugated secondary antibodies and stained with DAB. Two independent pathologists evaluated the staining score according to a method described previously (21).

### Statistical Analysis

The *t-test* was used to determine the statistical significance between groups with different expression levels of CXCL9. The *log-rank test* indicates the significance of survival time differences. Survival curves were automatically generated by the databases of Kaplan-Meier plots, GEPIA2, UCSC-Xena, HPA, TIMER, bc-GenExMiner, and GOBO. The median expression level of CXCL9 was determined to compare the survival outcomes, including distant metastasis-free survival (DMFS), disease-free survival (DFS), progression-free survival (PFS), relapse-free survival (RFS) and overall survival (OS), of breast cancer patients. A correlation analysis of gene expression was performed in TIMER. The analyses in the GEPIA2 databases and bc-GenExMiner database were performed using *Spearman’s correlation* analysis and *Pearson’s correlation* analysis, respectively. *P* values <0.05 were considered significant.

## Results

### The mRNA and Protein Levels of CXCL9 Were Markedly Higher in Breast Cancer Than in Normal Breast Tissue

**Figure 1:**
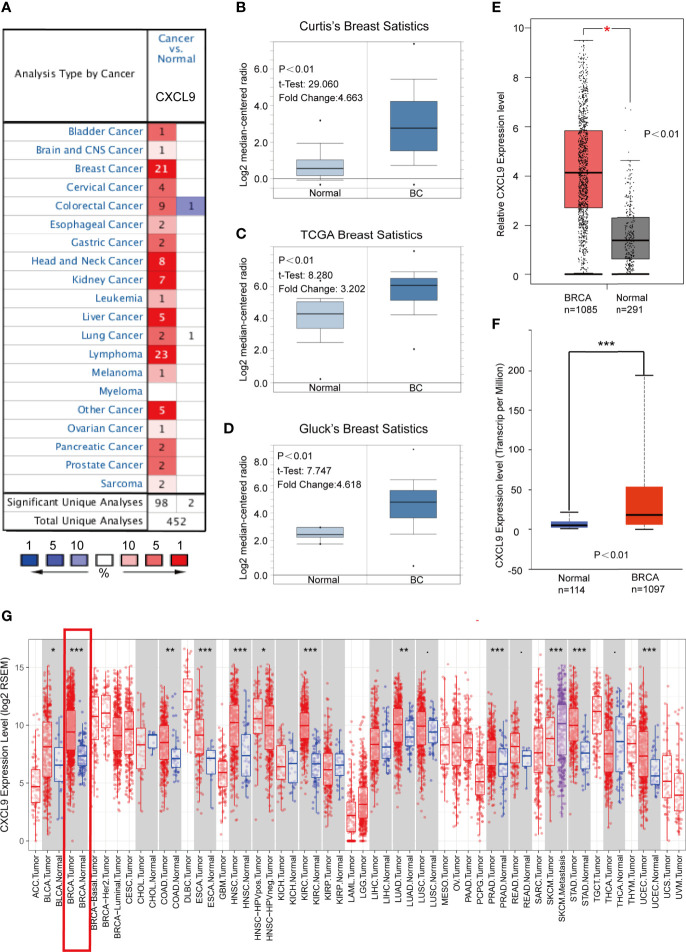
CXCL9 was significantly overexpressed in breast cancer compared with normal breast tissue. **(A)** Expression of CXCL9 (cancer *vs.* normal tissue) analysed with ONCOMINE database. The graphic demonstrated the numbers of datasets with statistically significant mRNA over-expression (red) or down-expression (blue) of the target gene. The p value threshold is 0.01. The number in each cell represents the number of analyses that meet the threshold within those analysis and cancer types. The gene rank was analysed by percentile of target gene in the top of all genes measured in each research. Cell colour is determined by the best gene rank percentile for the analyses within the cell. **(B–D)** Comparison of CXCL9 expression in Curtis’s Breast Dataset **(B)**, TCGA Breast Dataset **(C)** and Gluck’s Breast Dataset **(D)** from ONCOMINE. **(E)** The mRNA expression of CXCL9 in BC tissues from GEPIA2 database. **(F)** The expression of CXCL9 in BC tissues from UALCAN database. **(G)** Expression of CXCL9 (cancer *vs.* normal tissue) analysed among different tumor types from the TCGA in TIMER database (shown in red frame). The t-test was used to estimate the significance of difference in gene expression levels between groups. *P < 0.05, **P < 0.01, ***P < 0.001.

To determine the expression features of CXCL9 in breast cancer, we first compared the differences in CXCL9 expression between breast cancer and normal tissues through comprehensive analyses across multiple databases. The gene expression analysis using the Oncomine database showed that the mRNA levels of CXCL9 were significantly higher in BC than in normal tissues across an array of datasets in multiple cancer types (**Figure 1A**). In Curtis’s breast database, CXCL9 was elevated 4.663-fold in breast cancer compared with normal tissues in a dataset with 2,136 samples (**Figure 1B**, *P*<0.01) (22). Another dataset from The Cancer Genome Atlas (TCGA) demonstrated that CXCL9 transcripts were elevated 3.202-fold in breast cancer tissues compared with normal counterparts (**Figure 1C**, *P*<0.01). A similar result was found from a dataset from Gluck’s study (23), which showed that CXCL9 was increased 4.618-fold in breast cancer *versus* normal samples (**Figure 1D**, *P*<0.01). Data employed from Sorlie’s breast (24), Perou’s breast (25) and Karnoub’s breast (26) in the Oncomine database showed the same trend (**Supplementary Figures S1A–C**).

Similarly, analyses in both the GEPIA2 (with 1,085 tumour samples and 291 normal samples) and UALCAN (with 1,097 tumour samples and 114 normal samples) databases showed lower CXCL9 expression in normal tissues (**Figures 1E, F**). Moreover, the results of the TCGA RNA-seq data analysis using the TIMER and UALCAN databases indicated that CXCL9 mRNA expression was drastically higher in BC than in normal tissues among a wide range of tumour types (**Figure 1G** and **Supplementary Figure S1D**). These results demonstrated a consistent tendency of CXCL9 mRNA expression being elevated in BC tissues.

### CXCL9 Was Highly Expressed in ER-, PR- and Basal-Like Subgroups of BC Patients

**Figure 2:**
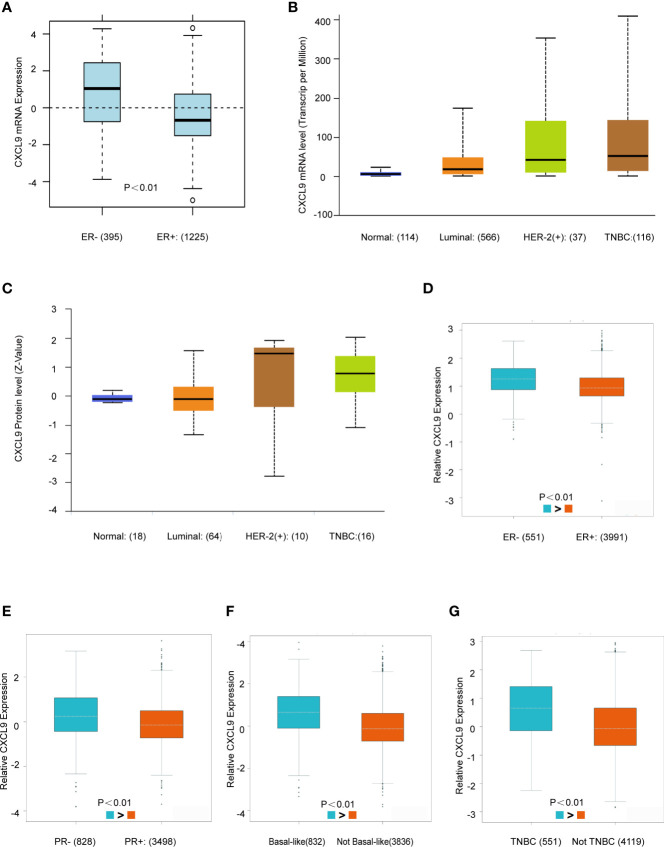
CXCL9 was highly expressed in ER-, PR- and Basal-like subgroups of BC patients. **(A)** The mRNA expression level of CXCL9 in ER negative and ER positive breast cancer subgroups in GOBO database. **(B, C)** The mRNA expression and protein level of CXCL9 among different subtypes of BC in UALCAN database. **(D–G)** The mRNA expression level of CXCL9 in BC patients with ER negative and ER positive **(D)**, PR negative and PR positive **(E)**, basal-like and non-basal-like **(F)**, TNBC and non-TNBC **(G)** from bc-GenExMiner sub-group analysis. The t-test was used to estimate the significance of difference in gene expression levels between groups.

Next, the GOBO, UALCAN and bc-GenExMiner databases were used to investigate the correlation between CXCL9 expression in disparate subpopulations of BC patients. The gene expression analysis derived from the GOBO expression module revealed that CXCL9 expression was distinctly higher in patients with ER-negative status (**Figure 2A**, *P*<0.01). Moreover, in the subgroup analysis of molecular subtypes using the UALCAN database, we found that both the mRNA and protein levels of CXCL9 in the TNBC subtype were markedly higher than those in the luminal subtype of BC (**Figures 2B, C**, *P*<0.01; **Supplementary Tables S1, S2**, *P*<0.01). In addition, the further subgroup analysis in the bc-GenExMiner database consistently showed elevated transcription levels of CXCL9. The results suggested that CXCL9 expression was concordantly higher in ER-negative, PR-negative, basal-like and TNBC samples than in their control counterparts (**Figures 2D–G**, *P*<0.01).

### Elevated Levels of CXCL9 mRNA Are Associated With Favourable Prognosis in Breast Cancer

**Figure 3:**
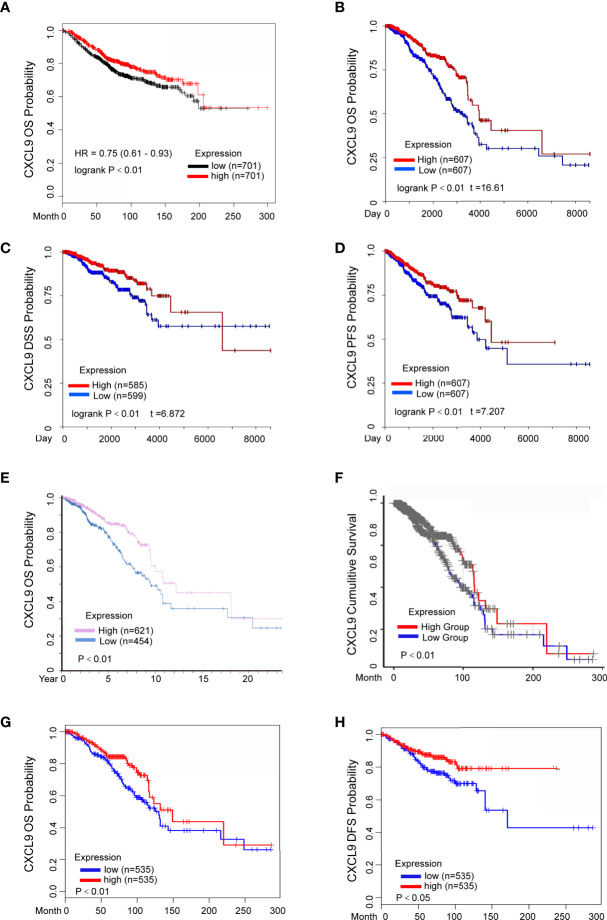
Elevated CXCL9 expression predicted better survival status in BC patients. **(A)** Overall survival estimates for CXCL9 mRNA levels from Kaplan-Meier plotter database. **(B–D)** Overall survival **(B)**, disease specific survival **(C)**, progression free survival **(D)** estimates for CXCL9 mRNA levels from UCSC Xena database. **(E)** Overall survival estimates for CXCL9 mRNA levels from Human Protein Atlas database. **(F)** Cumulative survival estimates for CXCL9 mRNA levels from TIMER database. **(G, H)** Overall survival **(G)**, disease free survival **(H)** estimates for CXCL9 mRNA levels from GEPIA2 database.

The prognostic value of CXCL9 in breast cancer was evaluated using the Kaplan-Meier Plotter, Human Protein Atlas, UCSC Xena, TIMER and GEPIA2 databases. The results from the Kaplan-Meier Plotter demonstrated that low CXCL9 expression was correlated with shorter OS in breast cancer (**Figure 3A**, HR=0.75, *P*<0.01), which was consistent with the results of OS (**Figure 3B**, *P*<0.01), DFS (**Figure 3C**, *P*<0.01), and PFS (**Figure 3D**, *P*<0.01) in the UCSC Xena database. Moreover, the results from the Human Protein Atlas suggested that higher CXCL9 expression correlated with better OS prognosis in breast cancer (**Figure 3E**, *P*<0.01). The results based on TIMER also showed that the high expression group of CXCL9 in BC may have a better CS (**Figure 3F**, *P*<0.01). In addition, the analysis in GEPIA2 revealed that higher CXCL9 levels were related to longer OS (**Figure 3G**, *P*<0.01) and DFS (**Figure 3H**, *P*<0.05) in breast cancer. These results demonstrated that high CXCL9 levels are connected with favourable outcomes in patients with breast cancer.

### The Prognostic Value of CXCL9 Was Related to Hormone Receptor Status in Breast Cancer

**Figure 4:**
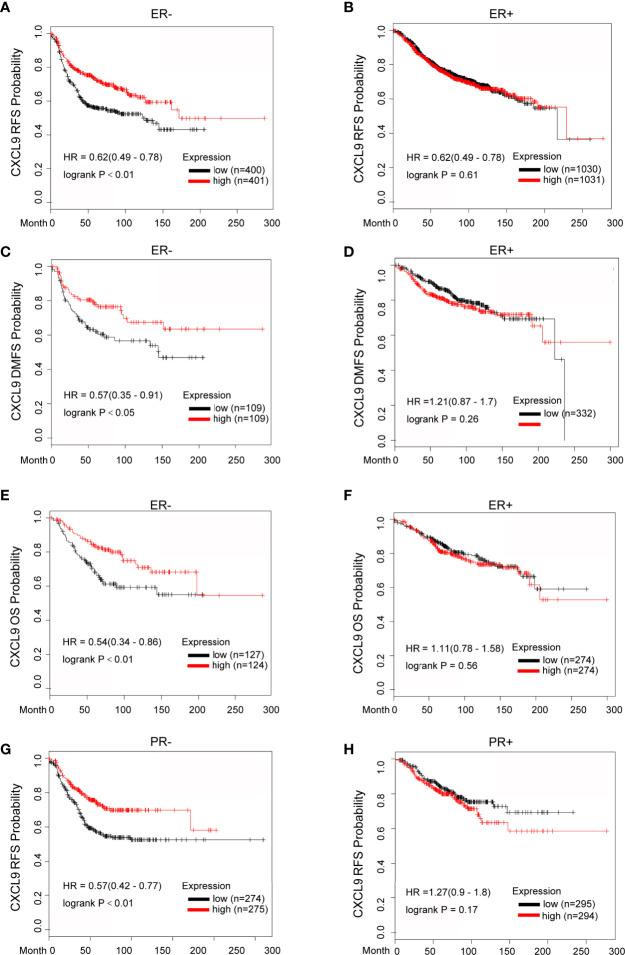
Elevated CXCL9 expression predicted favorable survival in distinct subtypes of breast cancer patients. **(A–F)** Recurrence free survival **(A, B)**, distance metastasis free survival **(C, D)**, Overall survival **(E, F)** estimates for CXCL9 mRNA levels in ER negative and ER positive subgroup BC patients. **(G, H)** Recurrence free survival estimates for CXCL9 mRNA levels in PR negative **(G)** and PR positive **(H)** subgroup BC patients.

To further investigate the prognostic role of CXCL9 in different subgroups of BC, we performed a correlation analysis according to hormone receptor status using the Kaplan-Meier Plotter, bc-GenExMiner and GOBO databases. The survival analysis from the Kaplan-Meier Plotter demonstrated that a higher mRNA level of CXCL9 was related to better RFS (Relapse free survival, **Figure 4A**, ER-, HR=0.62, *P*<0.01; **Figure 4B**, ER+, HR=0.62, *P*=0.61), DMFS (**Figure 4C**, ER-, HR=0.57, *P*<0.05; **Figure 4D**, ER+, HR=1.21, *P*=0.26) and OS (**Figure 4E**, ER-, HR=0.54, *P*<0.01; **Figure 4F**, ER+, HR=1.11, *P*=0.56) in BC patients in the ER-negative subgroup, whereas no significant difference was found in BC patients in the ER-positive subgroup. Moreover, BC patients with overexpression of CXCL9 presenting a PR-negative subgroup may have a longer RFS (**Figure 4G**, PR-, HR=0.57, *P*<0.01; **Figure 4H**, PR+, HR=1.27, *P*=0.17), while no significant difference was found in BC patients in the PR-positive subgroup.

Likewise, the results from bc-GenExMiner also showed that higher expression of CXCL9 was associated with better DMFS (**Supplementary Figures S2A, B**, ER-, HR=0.66, *P*<0.01; ER+, HR=1.06, *P*=0.59) and OS (**Supplementary Figures S2C, D**, ER-, HR=0.67, *P*<0.01; ER+, HR=1.10, *P*=0.08); in the ER-negative group, while there was no significance in patients with ER-positive tumours, which was consistent with the results from the GOBO database (**Supplementary Figures S2E–H**, DMFS: ER-, *P*<0.01; ER+, *P*=0.85; OS: ER-, *P*<0.01; ER+, *P*=0.48). These results suggest that the significant prognostic value of CXCL9 is mainly associated with oestrogen receptor status.

### The Expression Level of CXCL9 Was Associated With the Abundance of Immune Cell Infiltration in BC

**Figure 5:**
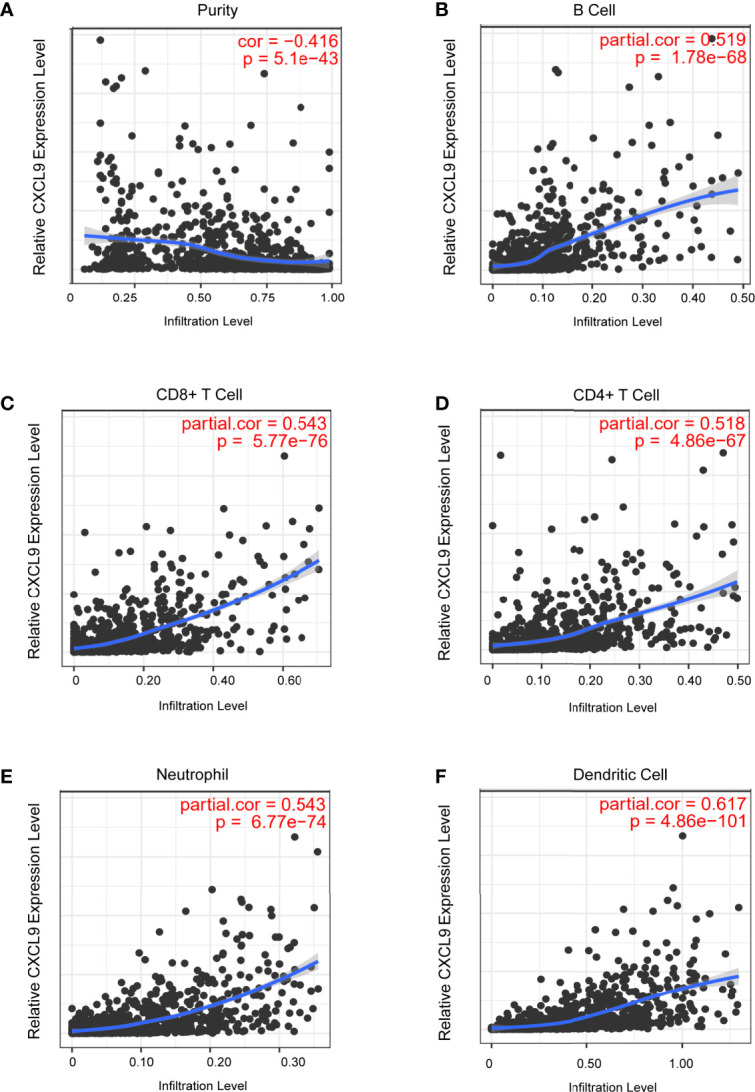
Correlation analysis of CXCL9 expression and infiltration levels of immune cells in BRCA tissues using the TIMER database. **(A–F)** CXCL9 expression in BRCA tissues positively correlates with tumor purity **(A)** and infiltration levels of B cell **(B)**, CD8+ T cell **(C)**, CD4+ T cell **(D)**, neutrophil **(E)**, and dendritic cell **(F)**.

Tumour-infiltrating lymphocytes (TILs) play a critical role in several cancers, including breast carcinoma. A previous study proved that the levels of quantity and activity status of TILs determine the survival time of patients with several cancers (27). Therefore, we explored the possible relationship between CXCL9 expression and TILs in BC, and the data in the TIMER database were analysed. CXCL9 expression was negatively correlated with tumour purity (**Figure 5A**, r=-0.416, *P*<0.01) in BC as well as its subtypes (**Supplementary Figure S3**). In addition, we observed that the level of CXCL9 expression was positively associated with the infiltration levels in BC: B cells (**Figure 5B**, r=0.519, *P*<0.01), CD8+ T cells (**Figure 5C**, r=0.543, *P*<0.01), CD4+ T cells (**Figure 5D**, r=0.518, *P*<0.01), neutrophils (**Figure 5E**, r=0.543, *P*<0.01) and dendritic cells (**Figure 5F**, r=0.617, *P*<0.01), which were consistent with the results of the subtype analysis (**Supplementary Figure S3**). These findings suggest a potential role of CXCL9 in modulating the infiltration of immune cells into the BC microenvironment.

### The Expression Level of CXCL9 Was Associated With Immune-Related Biomarkers

**Figure 6:**
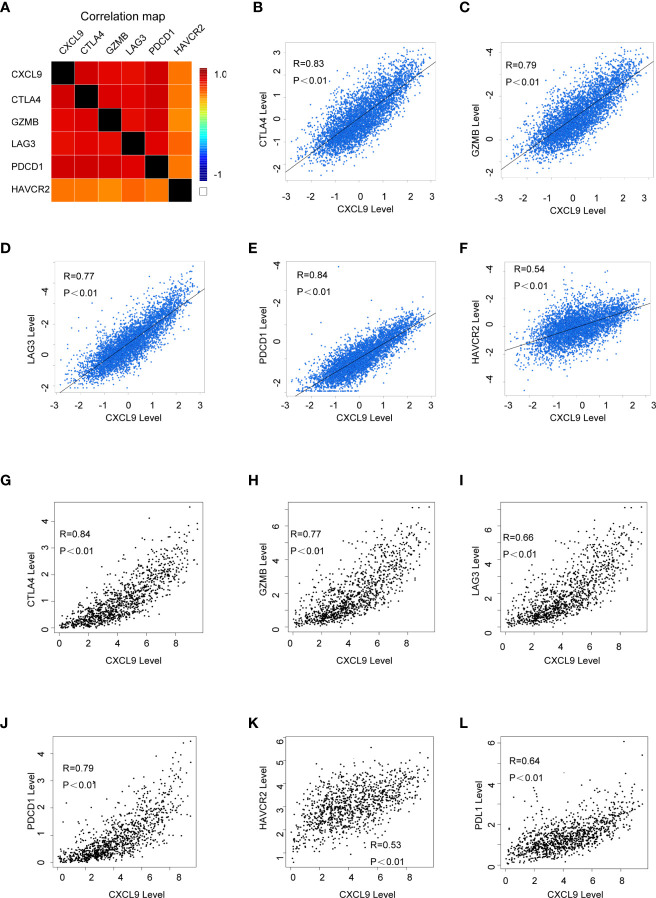
The correlation of CXCL9 expression and specific immune-related biomarkers in breast cancer. **(A)** Correlation heatmap of CXCL9 and targets of immunotherapy from bc-GenExMiner database. **(B–F)** Correlation analysis between the expression of CXCL9 and targets of immunotherapy from bc-GenExMiner database. **(G–L)** Correlation analysis between the expression of CXCL9 and targets of immunotherapy from GEPIA2.

In order to further probe whether CXCL9 is a potential biomarker for immune response for breast cancer, we examined the relationship between CXCL9 and a panel of recognized immune biomarkers and targets. Using the TIMER and GEPIA2 databases, we investigated the possible connection between CXCL9 and immune-related cells and their markers (the complete data are shown in **Supplementary Tables S3, S4**). The connection between CXCL9 and an array of genes involved in T cell exhaustion was also evaluated. A correlation heatmap of CXCL9 and specific T cell exhaustion genes from the bc-GenExMiner database was generated (**Figure 6A**). We observed that CXCL9 was positively and significantly correlated with CTLA4 (**Figure 6B**, r=0.83, *P*<0.001), GZMB (**Figure 6C**, r=0.79, *P*<0.01), LAG3 (**Figure 6D**, r=0.77, *P*<0.01), PDCD1 (**Figure 6E**, r=0.84, *P*<0.01), and HAVCR2 (**Figure 6F**, r=0.54, *P*<0.01); these results were basically in accordance with the results from the GEPIA2 database: CTLA4 (**Figure 6G**, r=0.84, *P*<0.01), GZMB (**Figure 6H**, r=0.77, *P*<0.01), LAG3 (**Figure 6I**, r=0.66, *P*<0.01), PDCD1 (**Figure 6J**, r=0.79, *P*<0.01), HAVCR2 (**Figure 6K**, r=0.53, *P*<0.01), and PDL1 (**Figure 6L**, r=0.64, *P*<0.01). Furthermore, according to previous studies, tumour purity plays a vital role in affecting the outcome of immune infiltration in a genomic manner (27). Therefore, the analysis consisting of tumour purity adjustment between CXCL9 expression and immune-related biomarker expression in BC tissues was completed using the TIMER database. The results from TIMER were consistent with the above findings (**Supplementary Figure S4**). These results further confirmed that CXCL9 may be related to tumour immune-related T cell exhaustion and may be a potential target for immunotherapy.

### CXCL9 Was Highly Expressed in ER-Negative Tumours and Positively Correlated With Immune-Related Factors, Including LAG3, PD1, PD-L1 and CTLA-4

**Figure 7:**
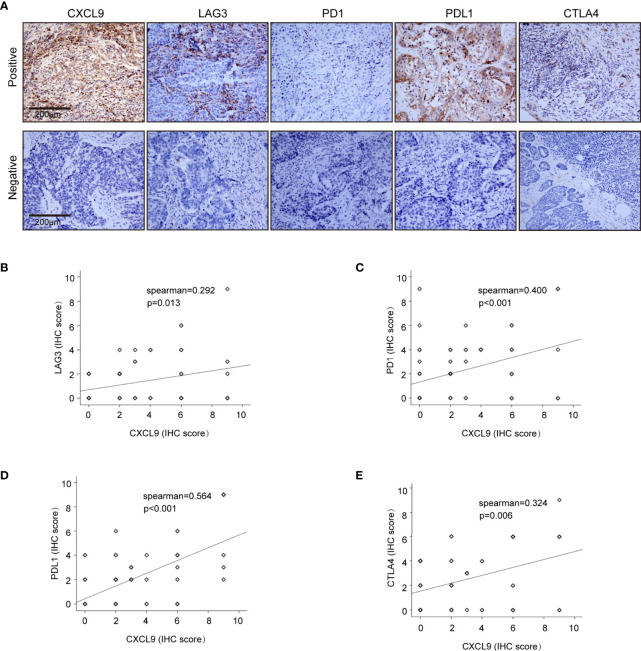
Correlation analysis of CXCL9 expression and immune infiltrating biomarkers in BRCA tissues. **(A)** Immunohistochemical staining for CXCL9, LAG3, PD1, PDL1, CTLA4. **(B–E)** Correlation analysis between the expression of CXCL9 and potential targets for immunotherapy LAG3, PD1, PDL1, CTLA.

To further validate our results in the bioinformatics analysis, we sought to determine the levels of CXCL9 in breast tumours and analyse its relevance to ER status, PR status and other clinicopathological features. We analysed a series of 115 patients with early stage breast cancer diagnosed in the Breast Centre, Cancer Hospital of Shantou University Medical College (SUMC) in 2016-2017. Through an immunohistochemistry (IHC) analysis (representative images shown in **Figure 7A**), we confirmed that CXCL9 expression was much higher in the ER-negative group compared with the ER-positive patients (**Table 1**, *P*=0.001). Likewise, the expression of CXCL9 was significantly lower in PR-positive patients (**Table 1**, *P*=0.012). Moreover, subgroup analysis revealed that CXCL9 was markedly lower in the Luminal subtypes than HER2 and TNBC subtypes (**Table 1**, *P*=0.01). To further investigate the connection between CXCL9 expression and the potential immune-response, the association with the expression of typical immune-related factors in BC patients was also analysed by IHC. Our results showed that elevated levels of CXCL9 positively correlated with the immune-related factors LAG3 (**Figure 7B**, r=0.291, *P*<0.05), PD1 (**Figure 7C**, r=0.400, *P*<0.01), PD-L1 (**Figure 7D**, r=0.564, *P*<0.01) and CTLA4 (**Figure 7E**, r=0.324, *P*=0.01).

**Table 1:** Correlation between CXCL9 expression and clinicopathological characteristics in patients with breast cancer.

Characteristics		CXCL9 (n = 115)		*P* value
		Low (n = 76)	High (n = 39)	
Age at diagnosis	≤50	47	25	0.813
	>50	29	14
Menopausal status	Premenopausal	28	15	0.865
	Postmenopausal	48	24
Tumour size	T0-T1	24	17	0.203
	T2-T4	52	22
Lymph node status	N0	36	18	0.902
	N1-N3	40	21
Stage	I	18	11	0.668
	II	39	21
	III	19	7
ER	Negative	21	23	0.001*
	Positive	55	16
PR	Negative	30	25	0.012*
	Positive	46	14
HER-2	Negative	60	27	0.25
	Positive	16	12
Ki67	≤30%	37	14	0.191
	>30%	39	25
Subtype	Luminal A	46	14	0.010*
	Luminal B	9	2
	HER2	7	10
	TNBC	14	13

*P < 0.05.

## Discussion

Immunotherapy had been used clinically against tumor metastasis, and studies had shown that metastasis could be prevented early by increasing the level of immune cells in primary tumors and circulation (28–30). However, due to immune escape from tumor cells, T cells are often insufficient for complete control of metastatic disease. Recently, a number of studies have demonstrated that the tumor immune microenvironment plays a crucial role in tumorigenesis and metastasis. Tumor infiltrating immune cells consist of the main regulators of tumor biology in the tumor microenvironment. In breast cancer, immunotherapy is one of the recent advances in cancer treatment, but the response rate is relatively low and varies with clinical subtype (30, 31). Currently, predictive biomarkers for immunotherapy remain limited, although pembrolizumab could be used in microsatellite instability-high breast cancers. However, evaluations of MSI-H as a predictor for single agent immunotherapy showed that the response rate is relatively low (32, 33), although the clinical interest is durable.

Chemokines are a subset of proteins that respond to G protein-coupled receptors. CXCL9, CXCL10 and CXCL11 constitute an important category of chemokines and are also structural ligands of the CXC-chemokine receptor 3 (CXCR3) receptor (34). Notably, the overexpression of tumour-derived CCL5 and myeloid cell-secreted CXCR3 ligands are crucial in manipulating CD8+ T cell infiltration in solid tumours (35). A study also revealed that CXCR3 ligands function as antiangiogenic molecules (36). Previous studies have demonstrated the orchestration of CXCR3 in the adoptive-transfer setting of effector T cells to tumours, indicating the pivotal role of chemokines in lymphocyte infiltration (37, 38). A tumour immune infiltration analysis by Yu Li revealed that CXCL9-11 was highly associated with natural killer (NK) cells and CD8+ T cells, may play a role in the antitumour immune response, and could be a novel prognostic biomarker in colorectal cancers (39). Recent studies highlight that the outstanding efficacy of anti-PD-1 therapy requires the upregulation of CXCR3 chemokine receptors on CD8+ T cells and CXCL9 produced by CD103+ dendritic cells (40, 41).

Our study demonstrated that the level of CXCL9 was associated with the abundance of immune cell infiltration in breast cancer, which partially validated the expression of CXCL9 in this particular cancer being related to changes in immune function. In particular, Zhao R et al. reported that the CXCL9/CXCR3 axis participated in the process of CD68 (+) CD163 (-) macrophages, thereby enhancing the efficacy of PD-L1/PD-1 blockades (42). Moreover, small cell lung cancer research revealed that the CDK7 inhibitor YKL-5-124 activated CXCL9 and that CXCL10 signalling may be a promising outcome of combining YKL-5-124 and anti-PD-1, confirming the pivotal function of CXCL9 in immunotherapy (43). The bioinformatics analysis results found that CXCL9 was positively correlated with the traditional immune function checkpoint markers CTLA4, GZMB, LAG3, PDCD1, HAVCR2, etc., while further immunohistochemical experiments also confirmed that CXCL9 expression in clinical tissue specimens was significantly correlated with LAG3, PD1, PDL1, CTLA4, etc. This implies that CXCL9 might exert a crucial function in the immune response of breast cancer.

A previous study proved that CXCL9 was significantly involved in lymphocytic infiltration in breast cancer that responded to neoadjuvant therapy (44). In particular, the concentration of CXCL9 was upregulated in oestrogen receptor (ER)-negative BC compared with normal cohorts (45). Moreover, in murine breast cancer models bearing CXCR3 and being induced by IFN-γ, CXCL9 was shown to have the ability to reduce tumour growth and metastatic colonization through recruitment of natural killer (NK) cells and activated T cells (10, 46), exhibiting a better prognosis in the CXCL9-overexpressing group (47). Razis et al. reported that high CXCL9 expression was a poor prognosticator for OS in breast cancer; however, it predicted favourable markers of both DFS and OS in subgroup patients with triple-negative disease (48). Similarly, through the integrated analysis of multiple database platforms, our data showed that elevated CXCL9 levels were associated with favourable prognosis in breast cancer, especially in ER-positive BC. This suggests that CXCL9 may be a predictor of prognosis in certain molecular subtypes of breast cancer. However, several studies have demonstrated that the CXCR3 axis provokes tumour growth and metastasis, and more in-depth investigation is needed to elucidate the CXCL9-related mechanism (49).

There are some limitations in this research. Firstly, most of the data investigated in this research were based on online databases, and related studies containing cell-based experiments, animal models, as well as extensive prospective clinical research are urgently needed in order to verify our results and to further investigate the potential mechanisms, and clinical values of CXCL9 in ER- breast cancer. Secondly, in our study, clinical specimens of breast cancer patients were used to verify the results of the previous bioinformatics analysis, which was a retrospective study on the survival and prognosis of breast cancer patients. Unfortunately, the number of breast cancer patients receiving immunotherapy is relatively small and the follow-up time for these patients is short. Therefore, the conclusion of this study is only an exploration of immune-related prognosis. The correlation between CXCL9 expression and immunotherapy needs to be further verified by immunotherapy related data. Finally, the current clinical trials of immunotherapy in the field of breast cancer are mostly focused on the exploration of advanced triple-negative breast cancer. Therefore, the availability of clinicopathological specimens is limited for prognostic prediction of early breast immunotherapy. However, at present, some clinical studies have shown that early triple negative breast cancer combined with immunotherapy can have good efficacy, so the significance of this study is to screen the subgroups that may benefit from immunotherapy in the early stage.

Overall, our findings suggest the potential role of CXCL9 as a predictor of immune infiltration in breast cancer. Our further study is ongoing to collect more cases of breast cancer patients treated with immunotherapy by testing CXCL9 expression in serological and paleohistological samples before and after treatment as well as exploring the molecular mechanism of CXCL9 in the modulation of breast cancer immunity.

## Conclusion

Consistent results from the analysis of multiple databases suggest that a higher level of CXCL9 is associated with longer survival and predicts an increased number of immune cells infiltration in ER-negative breast cancer. Future studies on the detailed molecular mechanism of CXCL9 in contributing to breast cancer immunity are warranted.

## Data Availability Statement

The datasets presented in this study can be found in online repositories. The names of the repository/repositories and accession number(s) can be found in the article/**Supplementary Material**.

## Ethics Statement

The studies involving human participants were reviewed and approved by the Ethics Committee of the First Affiliated Hospital of Shantou University Medical College. The patients/participants provided their written informed consent to participate in this study.

## Author Contributions

H-yL and DZ conceived and designed the project. Z-kD, M-tC, and S-qQ analysed the data and prepared the figures. Y-zQ, Z-hW, QX, S-cJ, and Y-sX performed the assays. Y-kL and Z-kD wrote the manuscript. H-yL and DZ approved the final version to be submitted. All authors contributed to the article and approved the submitted version.

## Funding

This study is partly supported by Interdisciplinary project of Li-Ka-Shing Foundation, (No.2020LKSFG05C & 2020LKSFG04A); Natural Science Foundation Committee (81901801), Natural Science Foundation of Guangdong Province (2021A1515011180 and 2019A1515010239), Guangdong Basic and Applied Basic Research Foundation (No. 2019A1515110953), General projects of Chinese Postdoctoral Science Foundation (2020M672753), “Dengfeng Project” for the construction of high-level hospitals in Guangdong Province –the First Affiliated Hospital of Shantou University Medical College Supporting Funding (No. 202003-5&202003-27) Youth Innovative Talent Project of Colleges and Universities in Guangdong Province, China (No.2019KQNCX035&2019KQNCX033). Shantou Municipal health science and technology project (No. 190819145262877); Science and Technology Special Fund of Guangdong Province, China (No. 190829105556145); Strategic and Special Fund for Science and Technology Innovation of Guangdong Province, China (No. 180918114960704); Guangdong Provincial Key Laboratory for Breast Cancer Diagnosis and Treatment (2017B030314116).

## Conflict of Interest

The authors declare that the research was conducted in the absence of any commercial or financial relationships that could be construed as a potential conflict of interest.

## Publisher’s Note

All claims expressed in this article are solely those of the authors and do not necessarily represent those of their affiliated organizations, or those of the publisher, the editors and the reviewers. Any product that may be evaluated in this article, or claim that may be made by its manufacturer, is not guaranteed or endorsed by the publisher.
