# Supplementary material for: CXCL9 Is a Potential Biomarker of Immune Infiltration Associated With Favorable Prognosis in ER-Negative Breast Cancer
Source: Front Oncol. 2021 Aug 30;11:710286. doi: 10.3389/fonc.2021.710286 (PMC8435794; doi:10.3389/fonc.2021.710286)
Supplement: Supplementary file 1 [file DataSheet_1.docx]

Supplementary Material

**Table S1 The mRNA expression of CXCL9 in TNBC subtypes was significantly higher than Luminal subtypes of BC**

| Group’s comparison | *P*-value |
| --- | --- |
| Normal *vs* Luminal | *P*<1.000×10^-12^* |
| Normal *vs* HER-2（+） | *P*=2.646×10^-4^* |
| Normal *vs* TNBC | *P*=9.117×10^-12^* |
| Luminal *vs* HER-2（+） | *P*=6.732×10^-2^ |
| Luminal *vs* TNBC | *P*=2.916×10^-3^* |
| HER-2（+） *vs* TNBC | *P*=8.376×10^-1^ |

* *P* < 0.05

**Table S2 The Protein level of CXCL9 in TNBC subtypes was significantly higher than Luminal subtypes of BC**

| Group’s comparison | *P*-value |
| --- | --- |
| Normal *vs* Luminal | *P*=2.675×10^-1^ |
| Normal *vs* HER-2（+） | *P*=2.290×10^-1^ |
| Normal *vs* TNBC | *P*=7.976×10^-3^* |
| Luminal *vs* HER-2（+） | *P*=3.465×10^-1^ |
| Luminal *vs* TNBC | *P*=3.404×10^-2^* |
| HER-2（+） *vs* TNBC | *P*=8.93×10^-1^ |

* *P* < 0.05

**Table S3** **Correlation analysis between expression of CXCL9 and relate genes and markers of immune cells in breast invasive carcinoma from TIMER**

| Immune cells | Gene markers | *P* | R |
| --- | --- | --- | --- |
| CD8+T cell | CD8A | 1.76E-208* | 0.785 |
|  | CD8B | 1.10E-121* | 0.653 |
| T cell (general) | CD3D | 4.20E-234* | 0.812 |
|  | CD3E | 1.17E-252* | 0.829 |
|  | CD2 | 2.05E-296* | 0.863 |
| B cell | CD19 | 1.44E-84* | 0.564 |
|  | CD79A | 7.73E-101* | 0.606 |
| Monocyte | CD86 | 1.44E-88* | 0.575 |
|  | CSF1R | 3.19E-21* | 0.294 |
| TAM | CCL2 | 3.64E-41* | 0.408 |
|  | CD68 | 1.80E-53* | 0.461 |
|  | IL10 | 1.73E-62* | 0.495 |
| M1 Macrophage | ISYNA1 | 5.21E-03* | -0.089 |
|  | IRF5 | 9.11E-17* | 0.26 |
|  | PTGS2 | 1.70E-03* | 0.099 |
| M2 Macrophage | CD163 | 2.26E-43* | 0.418 |
|  | VSIG4 | 4.80E-07* | 0.159 |
|  | MS4A4A | 1.68E-61* | 0.491 |
| Neutrophils | CEACAM8 | 7.16E-01 | 0.012 |
|  | ITGAM | 1.85E-23* | 0.309 |
|  | CCR7 | 9.06E-143* | 0.692 |
| Natural killer cell | KIR2DL1 | 1.93E-41* | 0.409 |
|  | KIR2DL3 | 8.35E-66* | 0.506 |
|  | KIR2DL4 | 1.27E-104* | 0.615 |
|  | KIR3DL1 | 3.44E-55* | 0.468 |
|  | KIR3DL2 | 2.56E-88* | 0.574 |
|  | KIR3DL3 | 6.10E-24* | 0.312 |
|  | KIR2DS4 | 1.53E-39* | 0.4 |
| Dendritic cell | HLA-DPB1 | 1.70E-74* | 0.534 |
|  | HLA-DQB1 | 2.58E-61* | 0.491 |
|  | HLA-DRA | 3.42E-129* | 0.667 |
|  | HLA-DPA1 | 2.61E-100* | 0.605 |
|  | CD1C | 5.57E-21* | 0.292 |
|  | NRP1 | 3.38E-02* | 0.067 |
|  | ITGAX | 1.01E-54* | 0.466 |
| Th1 | TBX21 | 2.93E-225* | 0.803 |
|  | STAT4 | 1.06E-134* | 0.678 |
|  | STAT1 | 8.75E-121* | 0.651 |
|  | IFNA1 | 7.45E-01 | -0.01 |
|  | TNF | 1.45E-20* | 0.289 |
| Th2 | GATA3 | 8.41E-10* | -0.193 |
|  | STAT6 | 3.94E-01 | -0.027 |
|  | STAT5A | 5.73E-02 | 0.06 |
|  | IL13 | 7.77E-15* | 0.243 |
| Tfh | BCL6 | 3.56E-01 | -0.029 |
|  | IL21 | 2.20E-73* | 0.531 |
| Th17 | STAT3 | 9.42E-01 | -0.002 |
|  | IL17A | 4.43E-13* | 0.227 |
| Treg | FOXP3 | 5.71E-206* | 0.782 |
|  | CCR8 | 3.99E-155* | 0.713 |
|  | STAT5B | 2.15E-01 | -0.039 |
|  | TGFB1 | 1.33E-03* | 0.102 |
| T cell exhaustion | PD-1 | 1.33E-167* | 0.732 |
|  | CTLA-4 | 2.92E-217* | 0.795 |
|  | LAG3 | 6.75E-125* | 0.659 |
|  | HAVCR2 | 1.87E-58* | 0.48 |
|  | GZMB | 2.00E-170* | 0.736 |

TAM: tumor-associated macrophage; Th: T helper cell; Tfh: Follicular helper T cell; Treg, regulatory T cell; Cor, R value of Spearman’s correlation，* *P* < 0.05

**Table S4：Correlation analysis between expression of CXCL9 and relate genes and markers of immune cells in breast cancer from GEPIA databases**

| Immune cells | Gene markers | *P* | R |
| --- | --- | --- | --- |
| Monocyte | CD86 | 2.60E−119* | 0.63 |
| Neutrophils | CD11b | 1.40E-38* | 0.38 |
|  | CCR7 | 3.90E-197* | 0.75 |
| TAM | CD68 | 8.40E-68* | 0.49 |
|  | IL-10 | 9.60E-78* | 0.52 |
| Th1 | IFN-γ | 3.80E-312* | 0.86 |
|  | STAT1 | 1.60E-127* | 0.64 |
|  | T-bet | 8.50E-295* | 0.84 |
|  | TNF-α | 8.00E-27* | 0.32 |
| Th2 | STAT6 | 4.50E-01 | 0.023 |
| Treg | CCR8 | 7.40E-183* | 0.73 |
|  | STAT5B | 8.20E-01 | 0.0069 |
|  | TGF-β | 4.40E-11* | 0.2 |
| T cell exhaustion | CTLA4 | 5.70E-286* | 0.84 |
|  | PD-1 | 7.30E-228* | 0.79 |
|  | TIM-3 | 3.90E-78* | 0.53 |

TAM: tumor-associated macrophage; Th: T helper cell; Tfh: Follicular helper T cell; Treg, regulatory T cell; Cor, R value of Spearman’s correlation，* *P* < 0.05


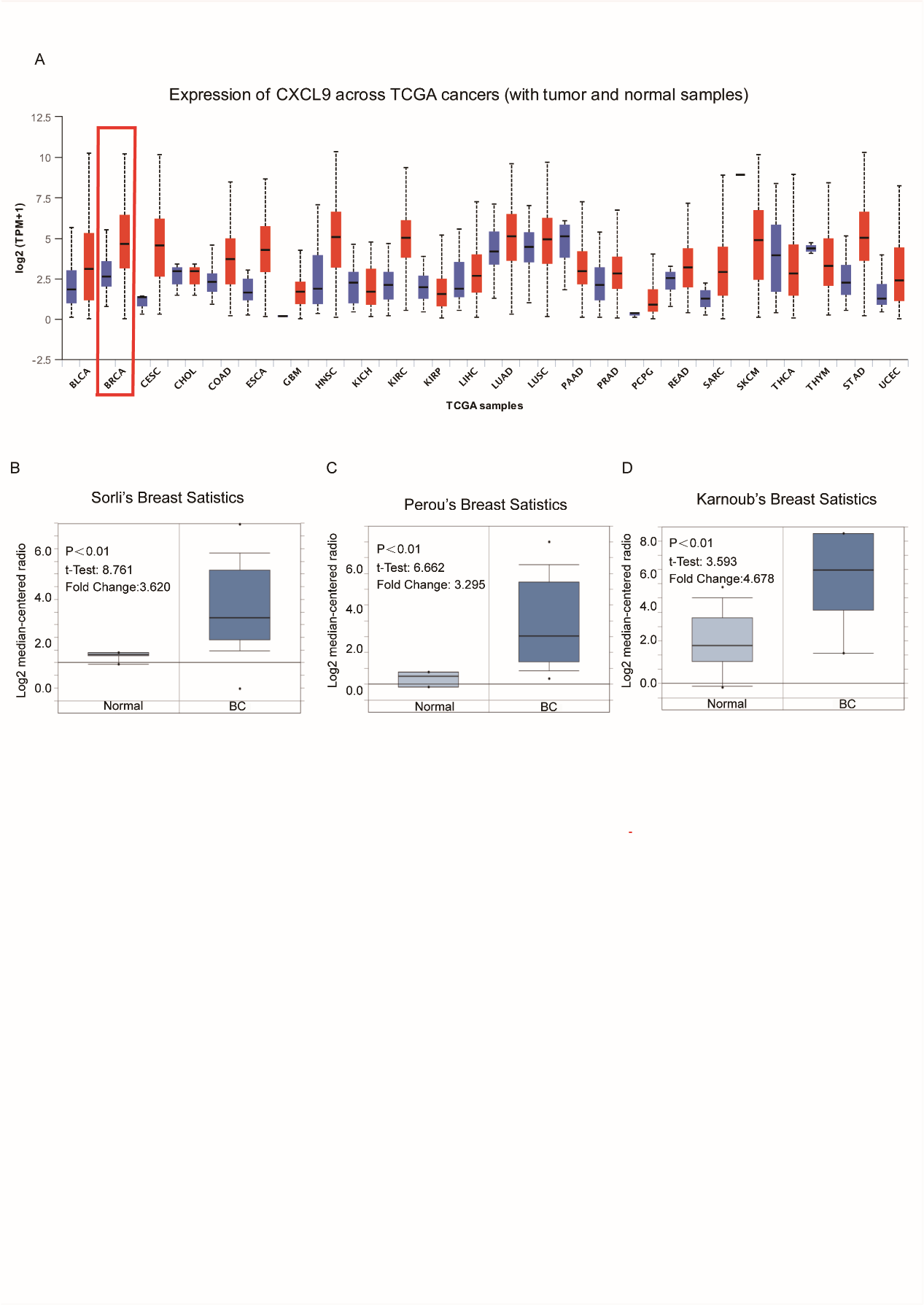


**Supplementary Figure 1.**. **CXCL9 was significantly overexpressed in breast cancer compared with normal breast tissue. (A-C)** Comparison of CXCL9 expression in Sorlie’s Breast Dataset(**A**), Perou’s Breast Dataset(**B**) and Karnoub’s Breast Dataset(**C**) from ONCOMINE database. **(D)** Expression of CXCL9 (cancer vs. normal tissue) analyzed among different tumor types from UALCAN database (shown in red frame). The t-test was used to estimate the significance of difference in gene expression levels between groups.


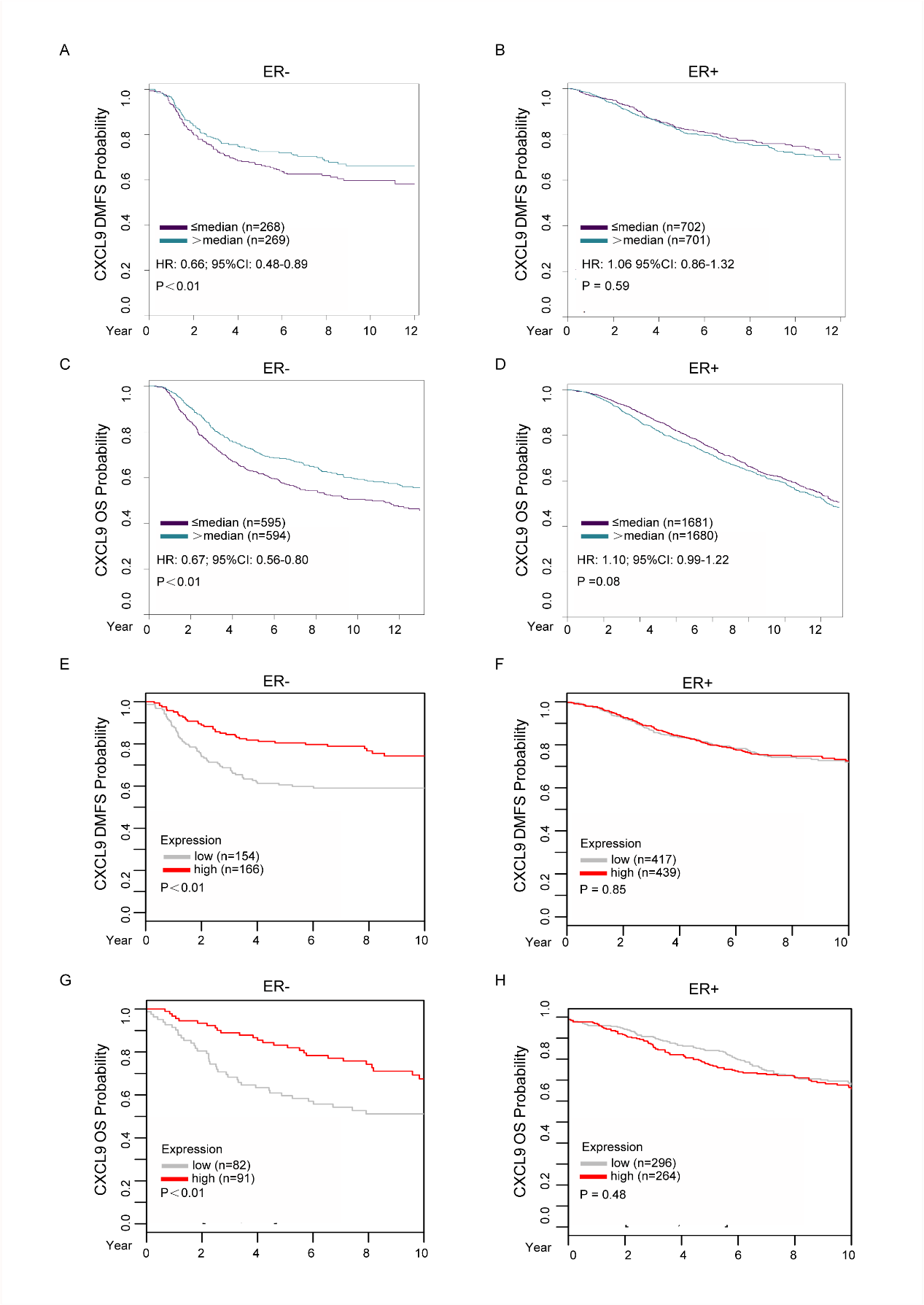


**Supplementary Figure 2.** **Elevated CXCL9 expression predicted favorable survival in distinct subtypes of breast cancer patients.** (**A-D**) Distant metastasis-free survival**(A-B)** and overall survival**(C-D)** comparation for the expression of CXCL9 in ER negative and ER positive subgroup BC patients from bc-GenExMiner database. (**E-H**) Distant metastasis-free survival(**E-F**) and overall survival(**G-H**) comparation for the expression of CXCL9 in ER negative and ER positive subgroup BC patients from GOBO database.


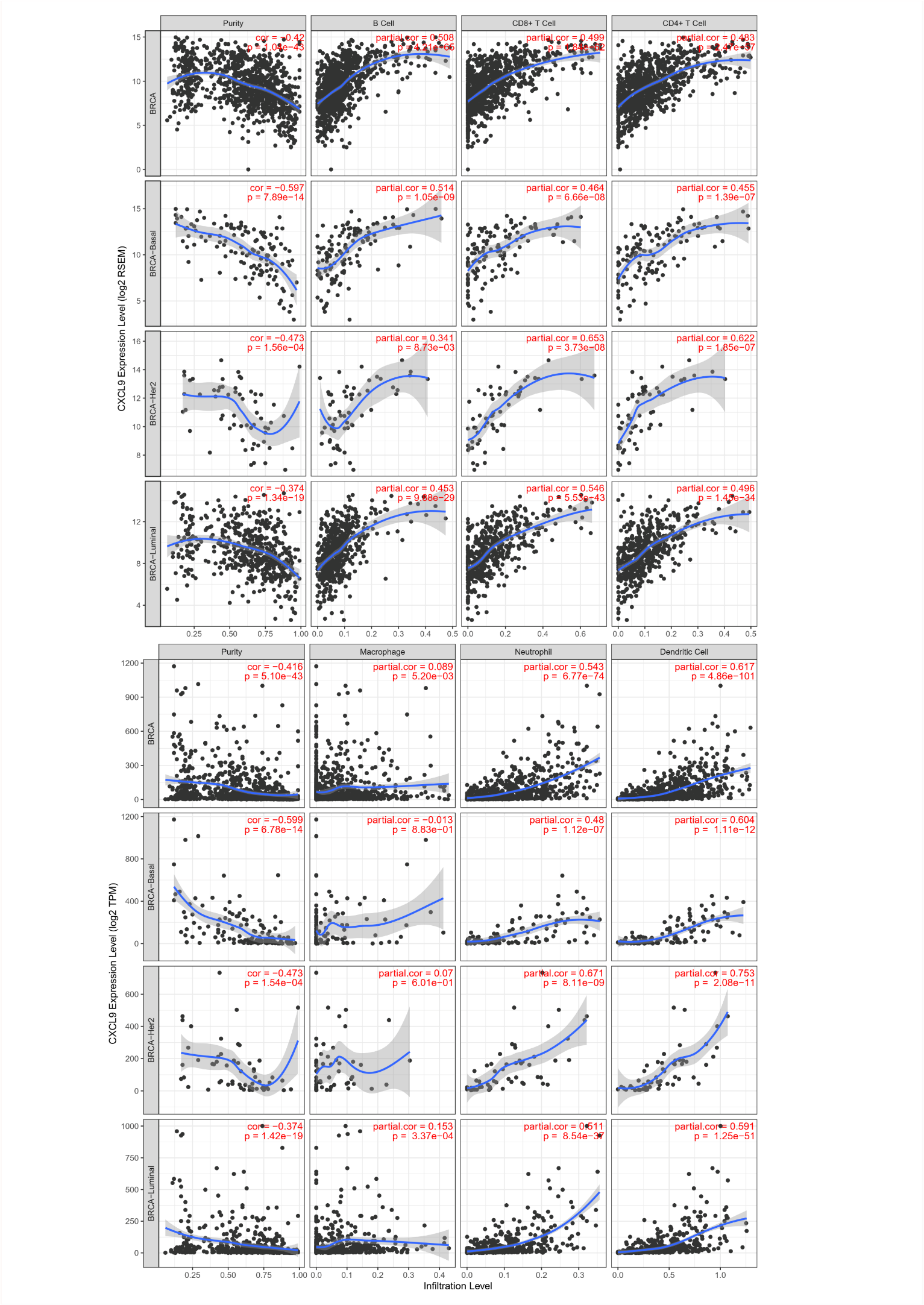


**Supplementary Figure 3.** **Correlation analysis of CXCL9 expression and infiltration levels of immune cells in different** **molecular subtypes of BC patients.** Correlation of CXCL9 expression in BRCA and different molecular subtypes of BC patients’ tissues with infiltration levels of B cell, CD8+ T cell, CD4+ T cell, Macrophage, neutrophil, and dendritic cell cells from TIMER database.


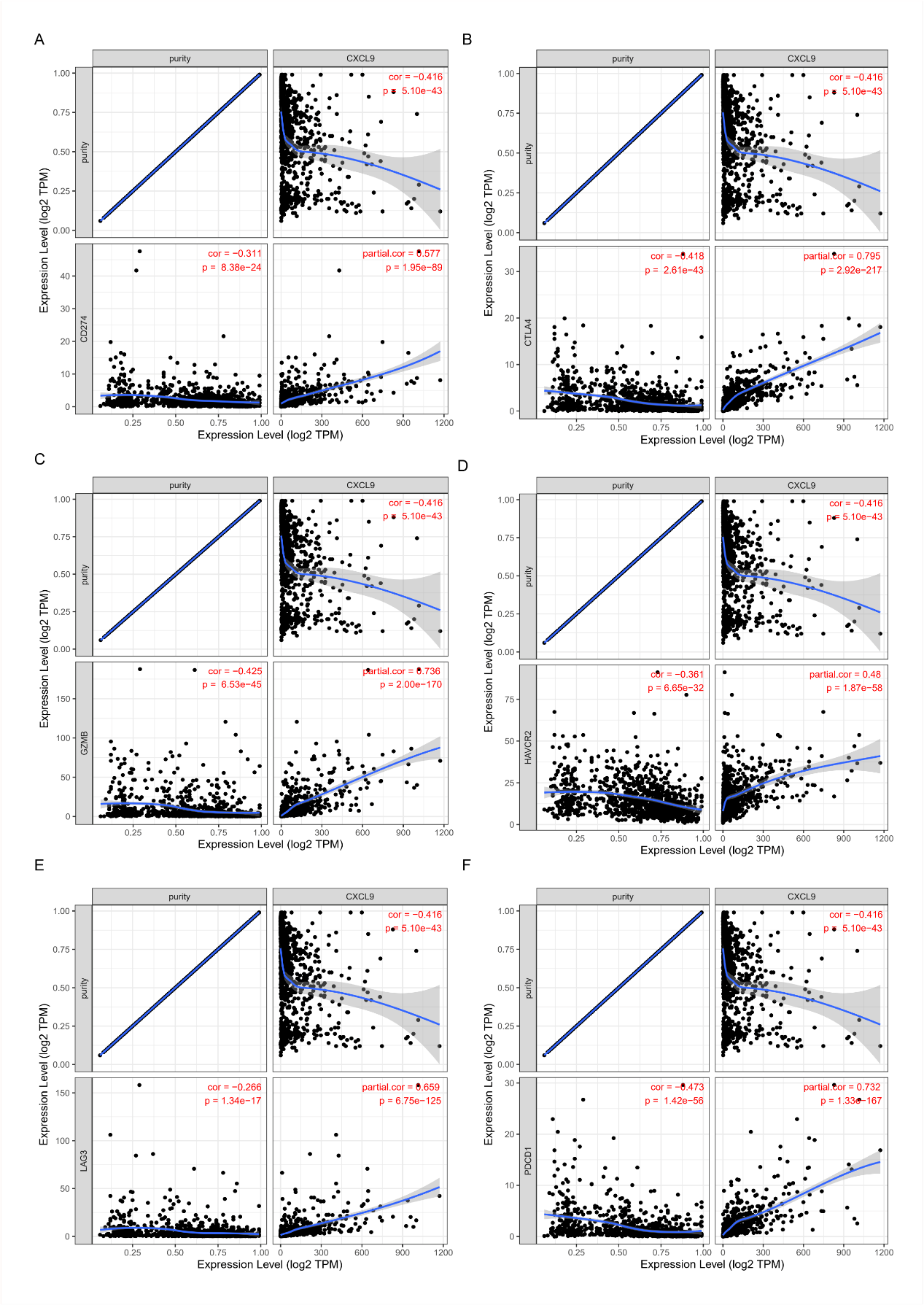


**Supplementary Figure 4. The correlation of CXCL9 expression and specific immune-related biomarkers in breast cancer**. **(A-F)** Correlation analysis between the expression of CXCL9 and targets of immunotherapy, CD274, CTLA4, GZMB, HAVCR2, LAG3 and PDCD1 from TIMER database
